# Supplementary material for: Influence of Electron Beam Irradiation on the Moisture and Properties of Freshly Harvested and Sun-Dried Rice
Source: Foods. 2020 Aug 19;9(9):1139. doi: 10.3390/foods9091139 (PMC7555959; doi:10.3390/foods9091139)
Supplement: Supplementary file 1 [file foods-09-01139-s001.zip › foods-884480-supplementary.docx]

Article

**Influence of electron beam irradiation on the moisture and properties of freshly harvested and sun-dried rice**

Lihong Pan^1, 2, 3, 4^, Jiali Xing^5^, Xiaohu Luo^1, 2, 3, 4*^, Yanan Li^1, 2, 3, 4^, Dongling Sun^6^, Yuheng Zhai^1, 2, 3, 4^, Kai Yang^1, 2, 3, 4^, Zhengxing Chen^1, 2, 3, 4*^

1 Key Laboratory of Carbohydrate Chemistry and Biotechnology, Ministry of Education, Jiangnan, Wuxi 214122, China; yelanplh@outlook.com (Lihong Pan); xh06326@gmail.com (Xiaohu Luo); jl2008010@outlook.com (Yanan Li); 6190112167@stu.jiangnan.edu.cn (Yuheng Zhai); yangkai164@outlook.com (Kai Yang); zxchen@jiangnan.edu.cn (Zhengxing Chen)

2 National Engineering Laboratory for Cereal Fermentation Technology, Jiangnan University, Wuxi 214122, China

3 Jiangsu Provincial Research Center for Bioactive Product Processing Technology, Jiangnan University, Wuxi, 214122, China

4 Collaborative Innovation Center for Food Safety and Quality Control in Jiangsu Province, Jiangnan University, Wuxi 214122, China

5 Ningbo Institute for food control, Ningbo 315048, China; hellojiali77@gmail.com (Jiali Xing)

6 Wuxi EL PONT Radiation Technology Co., Ltd., Wuxi 214151, China; 271559942@qq.com (Donglin Sun)

**Table S1.** Differences in proton areas and peak times among rice samples with different moisture contents.

| Sample | Control LM rice | Control LM rice-30 min | 4 kGy LM rice | 4 kGy LM rice-30 min | Control HM rice | Control HM rice-30 min | 4 kGy HM rice | 4 kGy HM rice-30 min |
| --- | --- | --- | --- | --- | --- | --- | --- | --- |
| Peak 1 | 739.12±3.47^b^ | 66.93±48.307^a^ | 850.37±17.72^c^ | 97.19±1.43^a^ | 817.94±11.47^bc^ | 97.60±48.25^a^ | 842.57±0.99^c^ | 81.52±3.49^a^ |
| Peak 2 | 18.47±0.28^a^ | 1236.67±17.85^b^ | 21.62±6.44^a^ | 1268.45±4.73^c^ | 14.14±5.27^a^ | 1310.41±13.42^d^ | 19.88±0.88^a^ | 1309.32±2.36^d^ |
| Peak 3 | 37.49±2.46^cd^ | 69.79±1.29^e^ | 45.54±5.56^d^ | 72.25±2.90^e^ | 22.50±0.75^ab^ | 39.47±3.77^cd^ | 20.65±1.06^d^ | 30.25±0.36^bc^ |
| T1 | 1.44±0.01^b^ | 0.51±0.10^a^ | 1.29±0.00^b^ | 0.59±0.01^a^ | 1.82±0.00^c^ | 0.47±0.00^a^ | 2.01±0.14^c^ | 0.60±0.02^a^ |
| T2 | 5.34±0.94^ab^ | 6.88±0.58^b^ | 3.23±0.60^a^ | 7.18±0.06^b^ | 7.30±2.24^b^ | 7.18±0.06^b^ | 4.90±0.16^ab^ | 7.18±0.06^b^ |

**Table S2.** Pasting properties of LM and HM rice samples under various irradiation doses. *

| Dose (kGy) | | Pasting properties | | | | |
| --- | --- | --- | --- | --- | --- | --- |
|  |  | PV (cP) | HV (cP) | FV (cP) | PT (°C) |  |
| LM rice | 0 | 2074.5 ± 21.5 ^a^ | 1135.0 ± 12.0 ^a^ | 1752.5 ± 14.5 ^a^ | 74.6 ± 0.4 ^ab^ |  |
|  | 1 | 1533.0 ± 3.0 ^b^ | 697.5 ± 9.5 ^b^ | 1120.0 ± 10.0 ^b^ | 74.2 ± 0.1 ^a^ |  |
|  | 2 | 1425.0 ± 2.0 ^c^ | 621.0 ± 9.0 ^c^ | 1002.0 ± 11.0 ^c^ | 75.0 ± 0.2 ^b^ |  |
|  | 3 | 1146.5 ± 8.5 ^d^ | 429.5 ± 0.5 ^d^ | 727.0 ± 1.0 ^d^ | 75.0 ± 0.1 ^b^ |  |
|  | 4 | 916.5 ± 1.5 ^e^ | 316.0 ± 0.0 ^e^ | 558.5 ± 0.5 ^e^ | 75.0 ± 0.2 ^b^ |  |
| HM rice | 0 | 2383.0 ±5.0 ^a^ | 1402.0 ± 6.0 ^a^ | 2003.5 ±5.5 ^a^ | 74.2 ± 0.1 ^a^ |  |
|  | 1 | 2021.0 ± 8.0 ^b^ | 1044.5 ± 6.5 ^b^ | 1586.0 ± 3.0 ^b^ | 75.1 ± 0.2 ^b^ |  |
|  | 2 | 1981.0 ± 4.0 ^b^ | 928.5 ± 1.5 ^c^ | 1452.5± 1.5 ^c^ | 74.7 ± 0.4 ^ab^ |  |
|  | 3 | 1477.5 ± 23.5 ^c^ | 655.5 ± 8.5 ^d^ | 1067.0 ± 9.0 ^d^ | 75.8 ± 0.2 ^c^ |  |
|  | 4 | 1234.0 ± 6.0 ^d^ | 472.5 ± 5.5 ^e^ | 798.5 ± 3.5 ^e^ | 76.7± 0.1 ^d^ |  |

* Mean value ± SD with different superscript letters in the same column are significantly different (p < 0.05).

**Table S3.** Thermal properties of flours prepared from rice grains treated with various irradiated doses. ^*^

| Dose (kGy) | | Thermal parameters^#^ | | | |
| --- | --- | --- | --- | --- | --- |
|  |  | T_o_ (°C) | T_p_ (°C) | T_c_ (°C) | *△H* (J/g) |
| LM rice | 0 | 62.62 ± 0.14 ^a^ | 68.13 ± 0.18 ^a^ | 74.13 ± 0.17 ^a^ | 9.50 ± 0.08 ^a^ |
|  | 1 | 62.36 ± 0.01 ^ab^ | 67.72 ± 0.05 ^b^ | 73.81 ± 0.13 ^a^ | 9.42 ± 0.09 ^a^ |
|  | 2 | 62.12 ± 0.07 ^b^ | 67.36 ± 0.09 ^b^ | 73.32 ± 0.03 ^b^ | 9.49 ± 0.00 ^a^ |
|  | 3 | 61.48 ± 0.07 ^c^ | 66.71 ± 0.09 ^c^ | 73.01 ± 0.01 ^b^ | 9.17 ± 0.28 ^a^ |
|  | 4 | 61.56 ± 0.03 ^c^ | 66.69 ± 0.08 ^c^ | 73.08 ± 0.08 ^b^ | 9.11 ± 0.08 ^a^ |
| HM rice | 0 | 62.37 ± 0.03 ^a^ | 67.55 ± 0.07 ^ab^ | 73.62 ± 0.06 ^ab^ | 9.53 ± 0.02 ^b^ |
|  | 1 | 62.07 ± 0.05 ^b^ | 67.63 ± 0.00 ^a^ | 73.79 ± 0.12 ^a^ | 10.93 ± 0.13 ^a^ |
|  | 2 | 61.84 ± 0.13 ^b^ | 67.23 ± 0.09 ^b^ | 73.73 ± 0.16 ^ab^ | 9.55 ± 0.01 ^b^ |
|  | 3 | 62.10 ± 0.05 ^bc^ | 67.37 ± 0.13 ^ab^ | 73.44 ± 0.12 ^ab^ | 9.30 ± 0.27 ^b^ |
|  | 4 | 61.47 ± 0.08 ^c^ | 66.90 ± 0.08 ^c^ | 73.35 ± 0.08 ^b^ | 9.12 ± 0.00 ^b^ |

* Mean value ± SD with different superscript letters in the same column are significantly different (p < 0.05).

# To, Tp, Tc, and ΔH represent onset gelatinization temperature (°C), peak gelatinization temperature (°C), conclusion gelatinization temperature (°C), and melting enthalpy (J/g), respectively.
